# Pandemic Depression: COVID-19 and the Mental Health of the Self-Employed

**DOI:** 10.1177/10422587221102106

**Published:** 2022-06-08

**Authors:** Marco Caliendo, Daniel Graeber, Alexander S. Kritikos, Johannes Seebauer

**Affiliations:** 1 University of Potsdam, IZA Bonn, DIW Berlin, IAB Nuremberg, Germany 122285; 2 SOEP at DIW Berlin, University of Potsdam, Germany 122285; 3 DIW Berlin, University of Potsdam, IZA Bonn, IAB Nuremberg, Germany 122285; 4 SOEP at DIW Berlin, Berlin School of Economics, Freie Universität Berlin, Germany

**Keywords:** Self-employment, COVID-19, mental health, gender, representative longitudinal survey data, PHQ-4 score, resilience, L26, D31, I14, I18, J16

## Abstract

We investigate the effect of the COVID-19 pandemic on self-employed people’s mental health. Using representative longitudinal survey data from Germany, we reveal differential effects by gender: whereas self-employed women experienced a substantial deterioration in their mental health, self-employed men displayed no significant changes up to early 2021. Financial losses are important in explaining these differences. In addition, we find larger mental health responses among self-employed women who were directly affected by government-imposed restrictions and bore an increased childcare burden due to school and daycare closures. We also find that self-employed individuals who are more resilient coped better with the crisis.

## Introduction

In 2020, governments around the world imposed strict lockdown policies aimed at containing the spread of SARS-CoV-2. These restrictions altered consumer behavior and disrupted demand in many sectors, changing the economic prospects, and decision-making of self-employed people in particular. Empirical analyses show that incomes of self-employed people in many countries have fallen since the beginning of the COVID-19 pandemic (Fairlie, 2020; Kalenkoski & Pabilonia, 2022). The self-employed have been more likely than employees to incur income losses, and self-employed women have been more severely affected by income losses than self-employed men (Graeber et al., 2021). This is a consequence of policy measures that are sensible from an epidemiological perspective (Bonacini et al., 2021), but threaten the survival of businesses, especially small and medium-sized enterprises.

The effects of the pandemic on self-employed people go far beyond economic losses. Initial descriptive evidence (Patel & Rietveld, 2020; Torrès et al., 2022) points to a worsening of mental health among the self-employed in the wake of pandemic-driven market distortions. Several reasons may explain these findings: first, self-employed people’s mental health may have suffered due to income losses (Gorgievski et al., 2010). Second, restrictions on business operations and opening hours reduced the working autonomy and decision authority of the self-employed, which may be detrimental to their mental health (Wiklund et al., 2019). Pandemic containment measures such as the closure of schools and daycare centers have forced self-employed parents to run their businesses while engaging in childcare and homeschooling, potentially increasing the burden on their mental health. Therefore, we investigate how the shock associated with the COVID-19 pandemic has affected the mental health of the self-employed, focusing particularly on gender differences. By using the wealth of information available in our data, we are able to gain a more complete picture of the multitude of factors affecting the mental health of self-employed people during the pandemic.

The relevance of our research question is underscored by the growing literature on the relationship between mental health and the decision-making processes of self-employed people (Wiklund et al., 2019). Mental health is an important component of human capital (Hatak & Zhou, 2021; Nikolaev et al., 2020; Torrès & Thurik, 2019) and human functioning (Ryan & Deci, 2001). Research shows that mental health systematically affects the decision-making and productivity of self-employed individuals. Depression can cause impairments in executive functioning, attention, and memory (Cobb-Clark et al., 2021; Hammar & Årdal, 2009). Individuals with major depressive disorders may engage in cognitive immunization processes, holding negative expectations and disregarding positive information that runs contrary to these expectations. This results in biased learning (Kube et al., 2020) and may lead to systematic underestimation of returns in cost-benefit analyses involving uncertainty. Cobb-Clark et al. (2021), for example, report a negative association between financial investments and being depressed.

In this study, we analyze the effect of the COVID-19 pandemic and related policy measures on the mental health of the self-employed. To isolate the effects on the self-employed from the effects on all working individuals, we use employees as a control group. Building on the well-established job demand-control (JDC) model, we predict a differential mental health impact of the COVID-19 pandemic on employed and self-employed people (see, e.g., Hessels et al., 2017). This model posits that jobs can be described along two dimensions: job demand and job control (Karasek, 1979). In a non-crisis context, the JDC model predicts better mental health among the self-employed than among employees since higher levels of job control—which are typical for the self-employed—are positively associated with mental health (Hessels et al., 2017). In the pandemic context, government-imposed restrictions aimed at containing the spread of SARS-CoV-2 reduced the job decision latitude of the self-employed, implying, *ceteris paribus*, a decline in the mental health of self-employed people who were affected by the restrictions compared to employed and to self-employed people who were not affected. Complementary to this, we investigate whether differences in the economic impact of the COVID-19 pandemic were a mechanism underlying a differential mental health response.

After isolating the impact of the pandemic on the self-employed, we further shed light on factors that moderate the effects within the population of self-employed people: procedural utility and resilience. Procedural utility describes the gain individuals derive from the way outcomes are generated (Benz & Frey, 2008a; Frey et al., 2004), thus explicitly linking mental well-being to the way the self-employed conduct their work. Given that the self-employed were restricted in their output, the associated loss of procedural utility might have negatively influenced their mental health. Resilience describes individuals’ ability to thrive if confronted with adversity (Gooding et al., 2012; Masten, 2002). Therefore, it may moderate the mental health response of self-employed individuals to the COVID-19 pandemic. In a similar vein, Anwar et al. (2023) find a positive relationship between resilience and organizational performance during the COVID-19 pandemic on the firm level. Evidence on resilience in times of crises is important since, if resilience is a mutable trait (for evidence pointing in this direction, see Williams, 2020), this opens up avenues for researchers and policymakers to learn about factors increasing individual resilience in the face of adversity.

Moreover, there is ample evidence that in many respects, the impact of the pandemic has not been gender-neutral. Women are often the primary caregivers in the family and are therefore disproportionately affected by the closure of schools and daycare centers (Alon et al., 2020). They are also more likely than men to reduce time spent in the labor market due to childcare (Couch et al., 2020). Notable gender differences in the labor market impact of the pandemic have also been documented for the self-employed (Fairlie, 2020; Kalenkoski & Pabilonia, 2022). This is explained to a large extent by the disproportionate sorting of self-employed women into industries that have been more strongly affected by the pandemic and associated government-imposed restrictions (Graeber et al., 2021), which may translate into a worsening of mental health. At the same time, Parslow et al. (2004), based on the JDC model, provide empirical evidence pointing to a positive relationship between work autonomy and mental health for self-employed women but not for self-employed men.

To test our hypotheses, we use a novel data set from the study “The Spread of the Coronavirus in Germany: Socio-Economic Factors and Consequences” (SOEP-CoV), which was launched in spring 2020 and interviewed the same individuals twice in 2020 and 2021. The survey included questions about respondents’ experiences during the COVID-19 pandemic, their work and family situations, and their attitudes (Kühne et al., 2020). What makes the SOEP-CoV data especially valuable for our purpose is that we can connect this information with the Socio-Economic Panel (SOEP), a representative longitudinal survey of German households that includes rich pre-pandemic information about demographics, socioeconomic characteristics, employment, personality, and other factors (Goebel et al., 2019). Importantly, SOEP-CoV includes the Patient Health Questionnaire-4 (PHQ-4) (Kroenke et al., 2009; Löwe et al., 2010), a well-established measure of the prevalence of symptoms of anxiety and depression, that is, mental distress, our main outcome of interest.

Estimating the causal effect of the COVID-19 pandemic on the self-employed is challenging. Time trends common to all working individuals could confound estimates between employment status and mental distress. These trends might include shocks on the macro level, such as exposure to SARS-CoV-2, and restrictions in the private domain, such as rules limiting social contact. Moreover, permanent differences in mental health could confound the estimates of interest. To overcome these challenges, we rely on a difference-in-differences (DiD) strategy, comparing the change in mental distress between self-employed and organizationally employed people over time. The resulting DiD estimates capture the effect of the COVID-19 pandemic on the self-employed only. Thus, comparing the self-employed to employees over time allows us to account for all consequences of the pandemic that were similar for all working individuals and to estimate the effect of those aspects of the crisis that were unique to the self-employed.

We find that the mental health of both the self-employed and employees worsened substantially and to similar extents during the pandemic. However, we uncover significantly larger changes in mental health among women. Differentiating by gender reveals that self-employed women experienced a stronger increase in the frequency of anxiety and depressive symptoms that was notably larger compared to self-employed men and employees. Moreover, the observed increases persisted throughout 2020, with 2021 survey results indicating only a slightly smaller magnitude. Furthermore, we find that the incidence of income losses is a predictor of mental distress among self-employed women, yet not among men. Our results also reveal that higher levels of resilience protect self-employed individuals from deteriorating mental health.

We further investigate possible explanations for the observed gender differences in mental health changes of the self-employed and find that being directly affected by government-imposed restrictions aimed at containing the spread of the virus is associated with significantly greater decreases in mental health among self-employed women, but find no effect for men. Similarly, having children living in the household and having increased childcare obligations as a result of pandemic restrictions translates into a worsening in mental health only for self-employed women.

We contribute to the literature on the effects of the pandemic on the self-employed in three ways: To the best of our knowledge, ours is the first study to investigate causal changes in the mental health of the self-employed in comparison to employees based on longitudinal data, thereby adding to the existing research on mental health and self-employment (Stephan, 2018). In contrast to studies based on cross-sectional data, our longitudinal study design enables us to control for time-invariant characteristics that may be simultaneously correlated with self-employment and mental health. Second, by focusing on gender differences, we contribute to the emerging literature on the gendered impact of the COVID-19 pandemic in general (e.g., Alon et al., 2020) and on gender differences in self-employment in particular (Fairlie, 2020; Graeber et al., 2021; Kalenkoski & Pabilonia, 2022). Third, we use information on government measures aimed at containing the spread of SARS-CoV-2 to provide a more holistic picture of the factors driving the mental health effects of the pandemic on the self-employed.

## Theoretical Background and Hypotheses

Self-employment offers both monetary and non-monetary benefits (Benz & Frey, 2008a) that lie at the core of the relationship between self-employment and mental health (Stephan, 2018). At the same time, good mental health is a crucial factor in business decisions and in the sustainability and growth of business undertakings, and it may be especially important for self-employed people (Hessels et al., 2018). In this section, we provide the theoretical and empirical background that guides our empirical analysis of the relationship between self-employment and mental health in the pandemic context.

### Differential Impact of the COVID-19 Pandemic on the Self-Employed and Employees

In the following, we discuss a potentially differential impact of the COVID-19 pandemic on self-employed and employed individuals. We focus on the well-established JDC model and on the role of income losses incurred as a result of the pandemic.

#### The JDC Model

A theoretical model that explains the association between self-employment and psychological distress is the JDC model. According to this model, jobs can be described along two dimensions: *job demands*, comprising work intensity, stress, conflicts, and workload, and *job control*, comprising subjective control over one’s work and skill development (Karasek, 1979; Theorell & Karasek, 1996). These two dimensions together determine the *job strain*, that is, the workplace psychosocial stress that is associated with the job, which, in turn, affects the mental health of the working individual. The JDC model predicts that a combination of high job demands and low job control results in *distress*, that is, negative stress, which is associated with worse mental health (Karasek, 1979; Theorell & Karasek, 1996). By contrast, individuals working in jobs with high job demand and high job control are expected to experience *eustress*, a positive form of stress, which can be beneficial for mental health (Hessels et al., 2017; Stephan & Roesler, 2010). Eustress allows individuals to learn and improve in their work and provides them with a feeling of mastery. Self-employed people typically have high job demands and high job control (Nikolova, 2019), whereas employees generally have lower decision latitude over the tasks they perform, implying lower levels of job control, all else being equal.

How the overall mental health of self-employed people compares to that of employees depends, according to the model, on the job characteristics. Empirical evidence shows that self-employed individuals report higher levels of decision authority (Parslow et al., 2004), which is linked to less work-related distress, compared to employees (Hessels et al., 2017). With respect to job demands, Parslow et al. (2004) find few differences between employment types. Accordingly, the majority of evidence concerning mental health differences between self-employed people and employees points toward a positive relationship between self-employment and mental health outcomes (e.g., Bradley & Roberts, 2004; Nikolova, 2019; Stephan & Roesler, 2010; Toivanen et al., 2016). One difficulty the literature faces is in establishing the direction of causality. Most research in this field seeks to estimate the effect of self-employment on mental health, but accounting for selection into self-employment is a nontrivial task (Rietveld et al., 2015; Stephan et al., 2020).^1^

Applied to the COVID-19 context, the JDC model suggests that the mental health impact of the pandemic on the self-employed depends on how individual *combinations* of job control and job demand have changed since the onset of the pandemic. Job control and job demands are likely affected by government interventions in the following way: government measures aimed at containing the spread of SARS-CoV-2 placed restrictions on business activities or forced businesses to temporarily shut down. In the short run, these measures left self-employed individuals with limited latitude and reduced decision authority, that is, control, over their business operations. How the pandemic affected the job demands of the self-employed is less clear. The government-imposed restrictions on businesses may have decreased their workload and work intensity. At the same time, the pandemic created new job demands: some self-employed people started rethinking their business models. Others applied for government aid, which involved substantial amounts of paperwork and bureaucratic effort. Businesses had to apply for bank loans to cover costs, and employers had to file for *Kurzarbeit*, that is, short-time work for their employees.^2^ In cases where self-employed individuals had to negotiate with creditors due to the financial challenges arising from the situation, conflicts sometimes arose. All of these factors are likely to change the job profiles of self-employed individuals. Ultimately, they may have led away from a more typical self-employed profile combining high job control and job demands, which is associated with eustress, to a profile of high job demands and low job control, thus creating distress, which is detrimental to mental health. This would also be in line with contemporaneous work from Backman et al. (2023), who show that the COVID-19 pandemic in Sweden negatively affected the well-being of self-employed individuals in terms of increased perceived stress.

For employees, predictions based on the JDC model are mixed. Some employees switched to remote work at the onset of the pandemic (Schröder et al., 2020; Zimpelmann et al., 2021), increasing their flexibility in deciding how and when to carry out their work, which would increase their job control. Others were placed on short-time work if their employer was suffering demand shortages. This would imply both a reduction in job demands and job control since opportunities for skill development were lacking. By contrast, employees in essential sectors (e.g., health care and grocery store workers) faced increasing job demands. Workload and time stress for these workers increased considerably during the pandemic (Prasad et al., 2021). In consequence, we would expect that, on average, the COVID-19 pandemic had a more attenuated effect on the mental health of employees relative to self-employed individuals.

#### Income and Mental Health

Changes in job control and demands are not the only way mental distress can increase. In this section, we elaborate on how we expect income losses to affect mental health and how these channels are moderated by employment status. We think of this as complementing the effect of changes in job demand and control.

Generating income from one’s own business may itself have effects on mental health. There is evidence that having a higher income from entrepreneurial activities improves mental well-being (for a review, see Stephan, 2018). In the context of the COVID-19 pandemic, the majority of self-employed people faced income losses from their business activities. In her overview, Stephan (2018) reports that the majority of studies on this topic find that financial losses are associated with a deterioration of mental health among the self-employed.

There are additional channels that could explain a relationship between income and mental health: (1) uncertainty associated with losses in income; (2) affordability of health inputs; and (3) interpersonal comparisons. One channel through which income could affect mental health is sustained uncertainty about income (Marin et al., 2011; Ridley et al., 2020). Notably, Patel and Rietveld (2020) find that financial uncertainty mediates the relationship between self-employment and mental distress in the United States during the COVID-19 pandemic. Thus, since self-employed individuals faced higher income uncertainty due to the crisis, we would expect higher mental distress among the self-employed relative to the employed.

Second, income may influence mental health by the affordability of health inputs (Grossman, 1972). These inputs may be nonmaterial, such as time, or they may be material market goods, such as appropriate health care (Grossman, 1972). In Germany, employees usually have statutory health insurance and are able to keep their health insurance even if they become unemployed. Self-employed people are typically insured through private health care providers. Therefore, while the income elasticity of demand for health care is practically zero for people who are not self-employed, it is high among the self-employed. This could mean a higher risk of mental health problems among the self-employed due to the lower affordability of health care. Finally, Ridley et al. (2020) argue that relative income could affect people’s mental health by way of social status or interpersonal comparisons.

We now turn to the question of how the economic consequences of the pandemic inform our predictions about its mental health impacts. Self-employed people were significantly more likely than employees to incur income losses that pose an existential threat to their businesses, potentially placing them under higher mental stress. In particular, Graeber et al. (2021) show that self-employed individuals in Germany were 42 percentage points more likely to report income losses resulting from the pandemic than employees, which may translate into negative mental health effects. Labor market rigidities and short-time work shielded employees from major income losses during the pandemic, whereas for the self-employed, demand shocks translate directly into income losses. And for employees who lost their job during the pandemic, the income shock is attenuated by unemployment benefits. The self-employed can opt in but are not required to have unemployment insurance.^3^

Therefore, based on predictions of the JDC model and considering the disproportionate economic impact of the pandemic on the self-employed and the lack of protective mechanisms for this employment group, we hypothesize that self-employed individuals experienced more negative changes in mental health than organizationally employed individuals:

*H1:* The COVID-19 pandemic had a more adverse mental health effect on self-employed people than on employees.

*H1a:* One important driver of this differential impact is the incidence of income losses following the pandemic.

### The Impact of the COVID-19 Pandemic on the Self-Employed: Moderators and Mechanisms

In the following, we discuss contextual factors that drive the differential mental health effects of the COVID-19 pandemic on the self-employed. These factors include pre-pandemic levels of procedural utility derived from business activities, levels of resilience, gender, and whether individuals were affected by government-imposed measures to contain the spread of SARS-CoV-2. Thus, we shift the focus to investigating changes in mental health within the population of the self-employed.

#### Procedural Utility

The active jobs hypothesis (high demand, high control) is closely related to the concept of “procedural utility” (Nikolova, 2019) insofar as job demands and control are important aspects of how people carry out their work. Specifically, procedural utility is the value people derive from the conditions and processes leading to material outcomes (Frey et al., 2004; Fuchs-Schündeln, 2009). Using items from the British Household Panel Survey (BHPS) that measure sources of procedural utility,^4^Benz and Frey (2008a) present evidence that the higher job satisfaction of the self-employed reflects procedural utility. Indeed, self-employed individuals have greater command over the type of work they perform and the way they perform it.^5^ In this context, Benz and Frey (2008a) show that self-employed individuals derive, on average, higher satisfaction from their work than employed individuals and that this is not driven by material aspects.

Another aspect of procedural utility is how useful individuals perceive their job to be (Benz & Frey, 2008b). Wolfe and Patel (2019) find that self-employed individuals tend to perceive their work as more useful for society than employees, and that this relationship is mediated by job satisfaction. From a mental health perspective, these aspects of procedural utility are negatively associated with mental health outcomes like depression, anxiety, and stress (Allan et al., 2018).

In this study, we will operationalize procedural utility in two different ways. We use respondents’ job satisfaction (Benz & Frey, 2008a) and their assessments of how useful and valuable they consider their work to be (Wolfe & Patel, 2019). The pandemic led to a major disruption in business operations. Many self-employed people were forced to temporarily shut down their businesses or were not able to offer their services. Considering this, we expect individuals who derive more procedural utility from their work to experience more mental distress, at least in the short run, if their ability to do business is impaired by government restrictions. We expect those who derive less procedural utility from their work to show less mental distress. Our expectations are based on the idea that self-employed people, who may derive higher levels of procedural utility from running their own businesses, are at greater risk of a loss of utility. If the level of procedural utility individuals derive is considered an endowment, this simply reflects loss aversion.^6^ Given the positive association between procedural utility and mental health, loss aversion implies a more negative mental health impact of the pandemic for individuals with larger endowments of procedural utility.

By contrast, one could argue that individuals who enjoy and feel fulfilled by their work or who perceive their job as particularly valuable will develop a strong drive to continue in the face of obstacles. They may try to make the best of a difficult situation. During the pandemic they may, for instance, seek different ways of doing business or new solutions that accommodate the circumstances at hand. However, government restrictions severely limited self-employed people’s options to adapt. Many were forced to stop providing services if their business was not judged essential. Thus, externally imposed restrictions on doing business, particularly mandated business closures, left many self-employed people little to no latitude to organize their own work. Therefore, we hypothesize:

*H2a:* The pre-pandemic level of job satisfaction moderates the negative effect of the COVID-19 pandemic on the mental health of self-employed individuals.

*H2b:* The pre-pandemic level of usefulness moderates the negative effect of the COVID-19 pandemic on the mental health of self-employed individuals.

#### Resilience

We are also interested in means that may have helped self-employed individuals to cope with the COVID-19 crisis. The concept of resilience describes the ability to thrive if confronted with adversity (Gooding et al., 2012; Masten, 2002) and is of considerable importance in the psychological literature (e.g., Bhamra et al., 2011; Fletcher & Sarkar, 2013; Masten, 2002). Resilience is used to explain people’s psychological responses in times of crisis. It is a multifaceted construct emphasizing the interdependencies among biological, psychological, and social processes in determining the ability to overcome adversity (Davydov et al., 2010). Psychologists differ in how they conceptualize resilience. Some consider resilience a trait, whereas others follow an outcome-oriented approach in which resilience is broadly defined as good functioning in the presence of adversity. In the latter approach, resilience is understood as a dynamic process involving an array of both risk and protective factors (Davydov et al., 2010; Kolar, 2011). Notably, Anwar et al. (2023) find that firms with higher levels of organizational resilience perform better during the COVID-19 pandemic than firms with lower levels of organizational resilience.^7^ Higher resilience may protect self-employed people’s mental health and may thus have improved their work performance during the COVID-19 pandemic. We therefore hypothesize that self-employed individuals who are particularly resilient, are better shielded from a decline in mental health due to the impact of COVID-19 on their businesses:

*H3:* Self-reported resilience attenuates the effect of the COVID-19 pandemic on the mental health of self-employed individuals.

#### Gender and Government-Imposed Restrictions

Self-employed women were about one-third more likely to experience income losses than their male counterparts during the first months of the pandemic in Germany. Much of this gender gap is explained by the disproportionate representation of women in industries that were more severely affected by the pandemic (Graeber et al., 2021), that is, the association between income losses and gender is mediated by industry affiliation.^8^ This mechanism arises as self-employed women tend to work in occupations that are directly affected by restrictions such as business closures. Recent research has identified another important driver of the gendered impact of the pandemic: the closure of daycare facilities and schools (Alon et al., 2020; Couch et al., 2020; Del Boca et al., 2020; Sevilla & Smith, 2020; Zamarro & Prados, 2021). Women are more likely to be primary caregivers (Arráiz, 2018). During the pandemic this means that self-employed women with children have been balancing increased childcare needs with the demands of running a business. It seems reasonable to assume that this multitude of demands triggers stress and is detrimental to mental health. This gives rise to the following mechanism: The pandemic led to the closure of schools and childcare facilities, which in turn presents a challenge to parents’ mental health. Women with young children are more likely to experience a deterioration in mental health because much of the childcare burden falls on them.

Both types of policy measures, the restrictions on doing business and the closures of schools and childcare facilities, reduce work autonomy and decision authority. In the context of the JDC model, the job control of self-employed women is more strongly affected than that of their male counterparts during the pandemic. Existing evidence further points to a more positive relationship between work autonomy and mental health for self-employed women than for self-employed men (Parslow et al., 2004). Moreover, Padkapayeva et al. (2018) show that women display higher levels of job strain than men. They also find that supervisor support—which self-employed individuals are typically lacking—matters more for women than for men. Finally, they show that job control is negatively associated with life stress for men, but not for women, and that job strain matters for women’s but not for men’s life stress. We therefore expect, on average, a stronger decline in mental health among self-employed women. We hypothesize:

*H4:* The expected disproportionate mental health effect of the pandemic on self-employed women is explained by their higher likelihood, relative to self-employed men, of working in businesses that were subject to pandemic restrictions.

*H5:* The expected disproportionate mental health effect of the pandemic on self-employed women is also explained by their higher likelihood, relative to self-employed men, of carrying the childcare burden arising from school and childcare closures.

## Background and Data

### The Data: SOEP-CoV

The SOEP-CoV survey was launched at the beginning of April 2020 to investigate the socioeconomic impacts of the COVID-19 pandemic in Germany.^9^ The same respondents were surveyed in a second wave at the beginning of 2021. Overall, 6,700 respondents were interviewed. Respondents were asked about their employment status, family situation, health, and attitudes during the COVID-19 pandemic (Kühne et al., 2020). The SOEP-CoV questionnaire includes a set of questions targeting the self-employed and a well-established measure of mental health and distress: the PHQ-4. Its integration into the SOEP makes the SOEP-CoV data well suited to our research question. The SOEP is a representative longitudinal survey of households in Germany that started in 1984 and is administered annually to households and the respective household members.^10^ As of 2020, the SOEP surveys approximately 20,000 households with more than 30,000 household members every year. The SOEP contains information about the household and about each household member, including economic, educational, attitudinal, and other variables (Goebel et al., 2019). Because SOEP-CoV represents a random subset of the SOEP population, it provides a rich set of pre-pandemic information on surveyed individuals.

For our main analysis, we focus on individuals who were either self-employed or employed (part- or full-time) in 2020^11^ and restrict our sample to observations from 2019 and 2020. For our longer-term analysis, we restrict the sample to observations from 2019 and 2021. In the first wave of the SOEP-CoV, the individuals sampled from the SOEP were surveyed between April and June 2020.^12^ The second wave of the SOEP-CoV survey was conducted in January and February of 2021. Our final sample for the first wave of the SOEP-CoV consists of 3,489 individuals. Our sample for the second wave of the SOEP-CoV, which we use to investigate long-term effects of the pandemic, includes 3210 individuals.

### Variable Description

**Figure 1.**
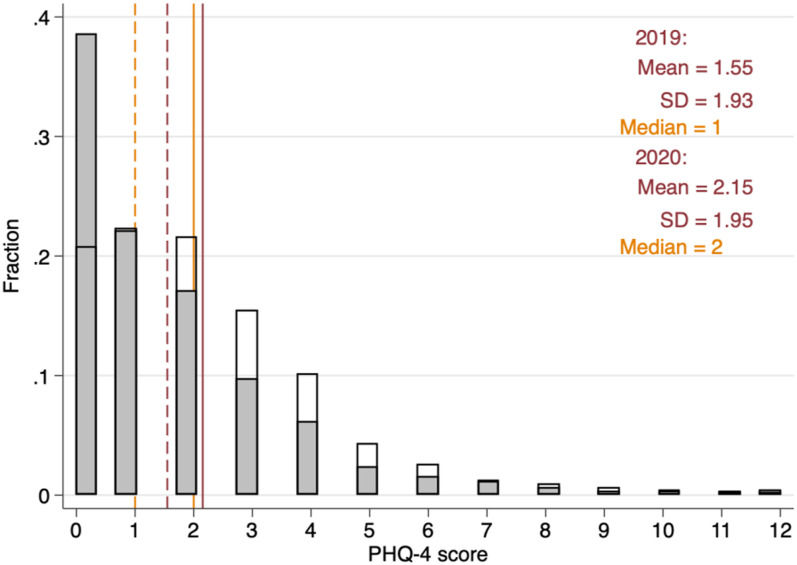
Distribution of the PHQ-4 score, 2020 (2019). *Note:* Figure 1 displays the distribution of the PHQ-4 score across the whole sample observed in 2020 (3489 individuals). The gray area shows the distribution for the same sample from 2019 for comparison. The red vertical line corresponds to the mean in 2020. The orange vertical line represents the 2020 median. The dashed lines show the corresponding statistics for 2019.

*Dependent variable:* We operationalize mental health by means of the PHQ-4 score, which is based on the PHQ-4 questionnaire. This questionnaire comprises a subset of the Patient Health Questionnaire that is used to screen for mental disorders. It is widely used in clinical assessment and epidemiological research (Löwe et al., 2010). The PHQ-4 score is the sum score of four items used to infer the frequency of symptoms of anxiety and depression (Kroenke et al., 2009) including “little interest or pleasure in doing things,” “feeling down, depressed, or hopeless,” “feeling nervous, anxious or on edge,” and “not being able to stop or control worrying.” Respondents state the frequency of these symptoms on a 4-point scale ranging from one “not at all” to four “(almost) daily.” The PHQ-4 score has been shown to be a valid and reliable measure of depression and anxiety in the general population (Löwe et al., 2010). We recode each item by subtracting 1, such that the absence of a symptom is associated with a value of zero. We then calculate the sum of all four items. Finally, for our analysis, we standardize the outcome to have a mean of zero and a standard deviation of one using 2019 as the base year. The distribution of the PHQ-4 score for the whole sample in 2020 is shown in Figure 1. For ease of comparison, the 2019 distribution is represented by the gray area. Notably, the mean PHQ-4 score strongly increased between 2019 and 2020, from 1.55 to 2.15, reflecting a general decrease in mental health. It is also striking that the incidence of depressive symptoms increased substantially. The mass at zero was roughly cut in half. While more than 38% of respondents in 2019 never experienced any symptoms of anxiety or depression, this was true for less than 21% in 2020. Moreover, the density increased substantially throughout PHQ-4 scores of 2–6, indicating a notable shift to the right in the frequency distribution of anxiety and depression symptoms.

**Figure 2.**
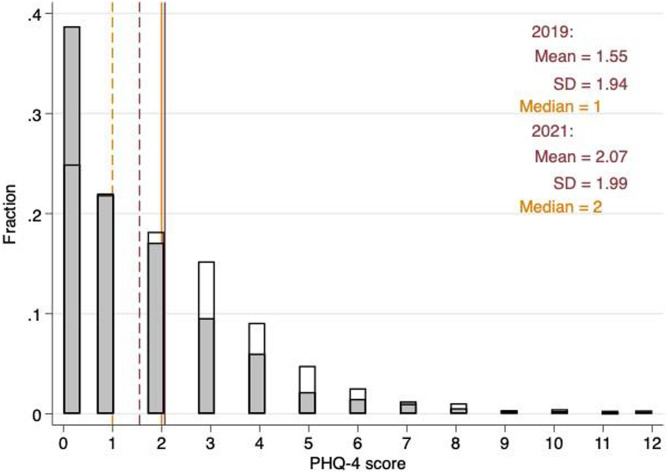
Distribution of the PHQ-4 score, 2021 (2019). *Note:* 2 displays the distribution of the PHQ-4 score across the whole sample observed in 2021 (3210 individuals). The gray area shows the distribution from 2019 for the same sample for comparison. The red vertical line corresponds to the mean in 2021. The orange vertical line represents the 2021 median. The dashed lines show the corresponding statistics for 2019.

For most individuals, we also observe their mental health scores in 2021. The corresponding distribution is shown in Figure 2. Both figures look strikingly similar, suggesting that the shift to the right in the frequency distribution of anxiety and depression symptoms observed in 2020 persisted into early 2021.

*Independent variables:* Our indicator for self-employment is based on information on respondents’ employment status in 2020. We label individuals who stated that they are working either full- or part-time in organizational employment as employees, and individuals who stated that they are self-employed as self-employed. We exclude individuals who stated that they are helping out self-employed family members. In the *Procedural Utility and Resilience* section of our analysis, we also employ two variables that partly govern changes in mental health during the pandemic: procedural utility and resilience. We measure procedural utility in two ways: through reported job satisfaction in 2019 and through the sense of purpose respondents reported feeling in their work.

Job satisfaction is measured with the question “How satisfied are you with your work?” Respondents answered on an 11-point Likert scale ranging from 0 “not satisfied at all” to 10 “completely satisfied.” Usefulness is measured with the question “Do you have the feeling that what you do in your life is valuable and useful?”. Again, respondents answered on an 11-point Likert scale ranging from 0 “not valuable and useful at all” to 10 “completely valuable and useful.”

**Figure 3.**
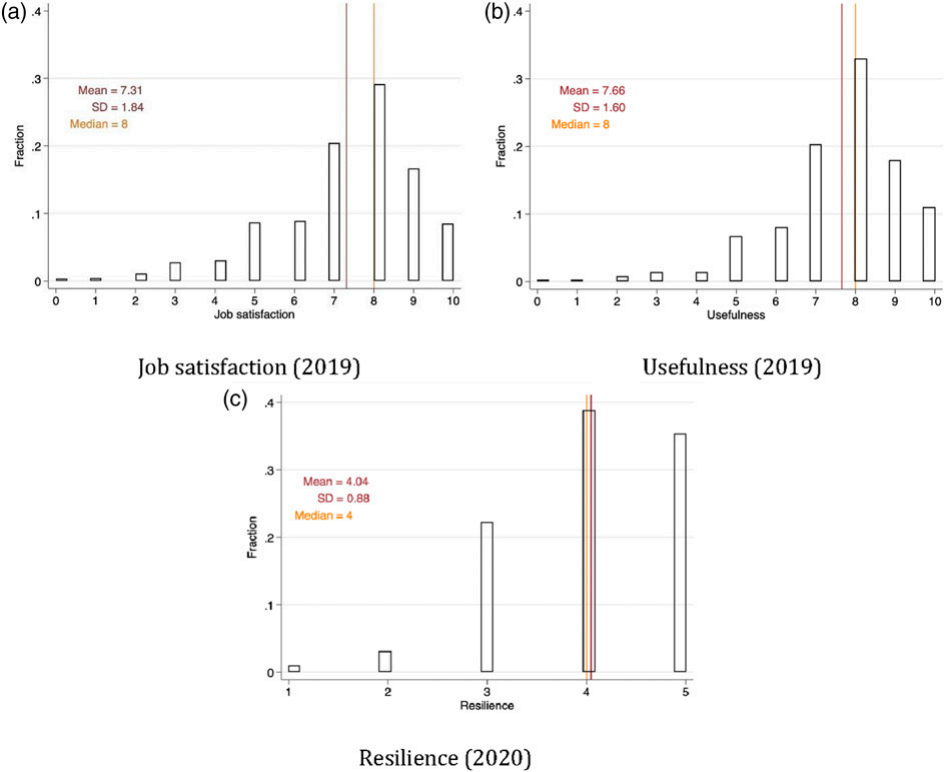
Distribution of job satisfaction, usefulness, and resilience. *Note:* Figure 3 displays the distributions of job satisfaction, usefulness, and resilience in the whole sample in 2019. The red vertical line corresponds to the mean. The orange vertical line represents the median.

Finally, resilience is measured by responses to the statement “I tend to recover quickly after difficult times.” Respondents answered on a 5-point Likert scale ranging from 1 “I disagree completely” to 5 “I agree completely.” For each of these three aforementioned concepts, job satisfaction, usefulness, and resilience, we perform a median split. The median for job satisfaction and for usefulness is eight. The median for resilience is four. The distributions of these three variables are displayed in Figures 3(a) to 3(c).

A further independent variable distinguishes respondents by whether they incurred income losses as a consequence of the COVID-19 pandemic. Employees were asked whether their gross earnings changed, and self-employed individuals were asked whether their income from self-employment changed. Thus, the framing of this question is causal.^13^ Respondents were asked to indicate whether their income “increased,” “decreased,” or “remained roughly the same.” Since few respondents indicated that their income increased, we construct a binary variable that takes the value 1 if the individual incurred losses in income, and zero otherwise.

*Summary statistics:*Table A1 provides a detailed description of our variables. Summary statistics for the year 2020 are shown in the Appendix, Table A2 and A.3.

To verify consistency of our measure of income losses with other data sources, we check whether the observed share of self-employed individuals experiencing income losses in the SOEP-CoV coincides with the general picture of income and revenue losses by comparing it to those from a report commissioned by the Federal Ministry for Economic Affairs and Energy. The authors of the report find that 61% of the surveyed firms reported a reduction in revenues in June 2020 (Federal Ministry for Economic Affairs and Energy, 2020). In our sample, 55% of respondents^14^ reported reductions in gross earnings over the period April to July 2020. Considering the slightly different timing of the two surveys, the different survey designs, and the fact that revenues and gross earnings do not necessarily map onto each other one-to-one, we conclude that these figures align well. Furthermore, 57% of self-employed respondents in SOEP-CoV also reported having experienced revenue losses (Kritikos et al., 2020). The corresponding 95% confidence interval in Kritikos et al.(2020) was [51%, 63%], including the point estimate of 61% in Federal Ministry for Economic Affairs and Energy (2020b). Therefore, we are confident that our sample indeed captures the dynamics in the underlying population.

### The COVID-19 Pandemic and Policy Measures in Germany

In January 2020, the first COVID-19 case was reported in Germany. In the following months, the number of COVID-19 cases increased steadily. The German government imposed severe restrictions beginning on March 22, 2020, just before the start of our sampling period. The associated non-pharmaceutical interventions included the closure of schools, daycare centers, restaurants, services in the field of personal hygiene, and most shops. Only essential businesses like grocery stores were allowed to remain open. In addition, public events were canceled, and the travel and hospitality sectors were heavily restricted. Public gatherings were limited to two individuals, and people were required to keep a distance of at least two meters from each other in public spaces (Federal Ministry of Health, 2020).

**Figure 4.**
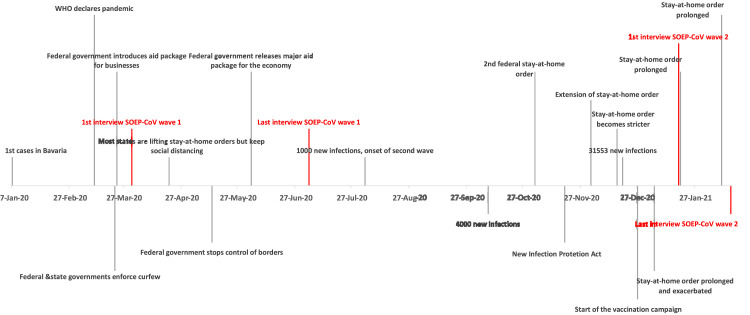
The policy measures in the wake of the COVID-19 pandemic over our sampling period. *Note:* Figure 4 displays the policy measures over our sampling period.

**Figure 5.**
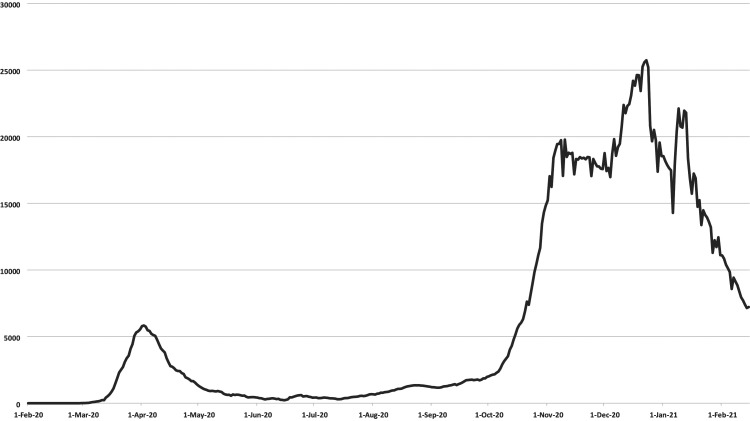
The COVID-19 pandemic over the sampling period. *Note:* Figure 5 displays the number of new COVID-19 cases. The number of COVID-19 cases is based on Ritchie et al. (2021).

During our period of observation between April and July 2020, the German government also introduced several policy measures to mitigate the economic consequences associated with the pandemic. The most relevant policy measure was *Kurzarbeit*, the short-time work compensation scheme in which the Federal Employment Agency (FEA) subsidized up to 67% of employees’ net income. As the self-employed were not covered by this scheme, the federal government released an emergency aid package providing up to 50 billion Euros to micro-enterprises. This ad-hoc program was aimed at supporting self-employed individuals who faced severe declines in revenues by offering lump sum payments of up to 15,000 Euros. However, its use was limited to covering fixed operating costs and could not be used to cover living expenses (Block et al., 2020). In addition, the self-employed received somewhat easier access to the *Arbeitslosengeld 2* unemployment benefits, (Federal Ministry for Economic Affairs and Energy, 2020a), but barriers remained high. A timeline of events and policies is depicted in Figure 4, the number of cases in Figure 5.

The second SOEP-CoV survey wave was administered in January and February 2021, during the second wave of the COVID-19 pandemic. The second wave of infections, as depicted in Figure 5, was more severe and widespread than the first wave. Policy measures for employees did not change over time. In the second half of 2020, policy measures targeting self-employed individuals were adapted based on previous revenues. The payments associated with “*Überbrückungshilfe”* or bridging aid was provided to keep directly affected businesses afloat. In contrast to the initial aid, bridging aid could be used by recipients to cover their private expenses exclusively in November 2020 (BMWi and BMF, 2021).

## Empirical strategy and Results

### Empirical Strategy

The COVID-19 pandemic and associated policy measures have a wide range of implications that are not specific to the self-employed (Goodman-Bacon & Marcus, 2020). We use employees as a control group to distinguish between the effects that are equal across all working individuals and the effects that only affect the self-employed, that is, the causal effect of the pandemic on self-employed individuals. Thus, following a difference-in-differences (DiD) approach, we estimate the following model:

(1) $$y_{it}=\beta_{0}+\beta_{1}Selfemp_{i}\cdot COVID_{t}+\zeta_{\iota }+\beta_{2}COVID_{t}+\in_{it}.$$

In Equation (1), *y*_*it*_ is our mental health measure, the PHQ-4 score. The indicator *Selfemp*_*i*_ is equal to one if individual *i* is self-employed and zero if the person is an employee. We partial out individual fixed effects *ζ*_*i*_ to account for individual-level differences that are due to time-invariant characteristics, that is, we de-mean our data. We thus account for various permanent differences between the self-employed and employees that would otherwise confound our estimate of interest. For instance, the self-employed and employees differ systematically along various dimensions that may jointly govern the selection into self-employment and mental health (Stephan et al., 2020). Examples of such permanent differences are education, migration background, and other socio-demographic characteristics. Table A3 shows that self-employed individuals are better educated and less likely to have a migration background than employees, on average. Importantly, in our setup, we account for these differences, even if relevant characteristics are unobserved in our data: if these characteristics are time-invariant, they are captured by the individual fixed effects. However, we do not control for any time-variant characteristics since these could themselves be affected by the treatment, that is, being self-employed during the COVID-19 pandemic compared to being employed, and could seriously confound the estimate of interest (Rosenbaum, 1984). Moreover, *COVID*_*t*_ is an indicator that is equal to one for observations during the COVID-19 pandemic, that is, for the year 2020 or 2021, depending on the analysis, and zero otherwise. This accounts for the common trend in mental health across groups. Contextual differences between 2019 and 2020 that are common to both groups could include, for instance, the risk of being infected with COVID-19 or policy measures that affect both the self-employed and employees.

Our design corresponds to a classic 2*x*2 DiD design with individual fixed effects. To identify the causal effect of being self-employed compared to being a regular employee, we require that the trend in the mental health outcome would have been comparable across groups in the absence of the COVID-19 pandemic. This is a counterfactual situation that requires knowledge of the alternate state, that is, the mental health of individuals if the COVID-19 pandemic had not occurred. It is therefore not testable. However, we find suggestive evidence for the validity of the common trend assumption by comparing the evolution of mental health between self-employed individuals and employees in the years before 2020, thus establishing common trends in the pre-COVID-19 period. To account for within-individual-level correlation of mental health over time, we cluster the standard errors at the individual level.

**Figure A2.**
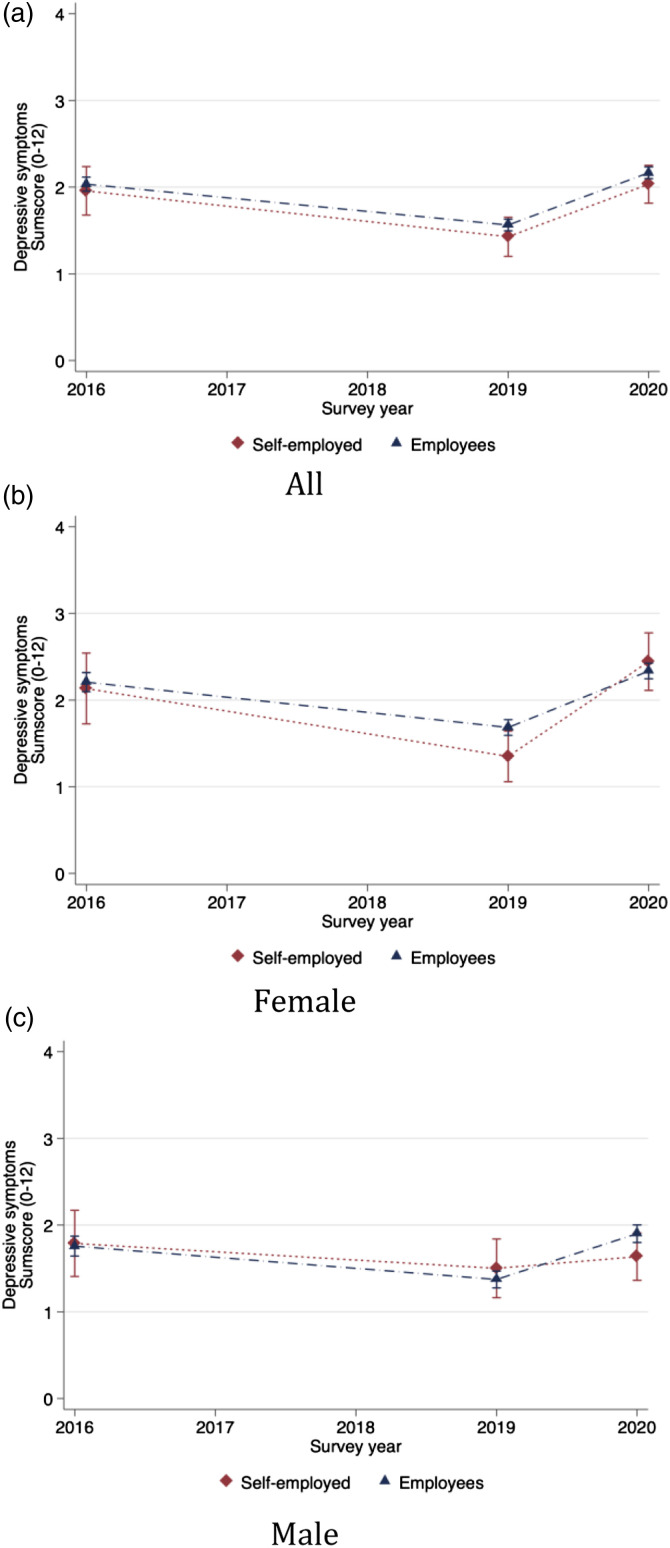
Differences in mental health between entrepreneurs and employees. (a) All, (b) female, and (c) male. *Note:* Figure A2 shows the evolution of the PHQ-4 score for different groups in the sample from 2016 to 2020. The whiskers around the point estimates correspond to the 95% confidence intervals.

Figure A2 compares the evolution of the PHQ-4 score between the self-employed and the employees over time. Between 2016 and 2019, prior to the COVID-19 pandemic, the PHQ-4 scores evolved in a parallel fashion, thereby lending support to the appropriateness of the common trend assumption. Thus, we are confident that the OLS estimate of *β*_1_ on the interaction of *Selfemp*_*i*_ and *COVID*_*t*_ represents the effect of being self-employed during the COVID-19 pandemic. To test hypothesis *H1*, we interact our treatment, being self-employed during the COVID-19 pandemic, with an indicator for experiencing income losses during the COVID-19 pandemic.

### Main Results

**Figure 6.**
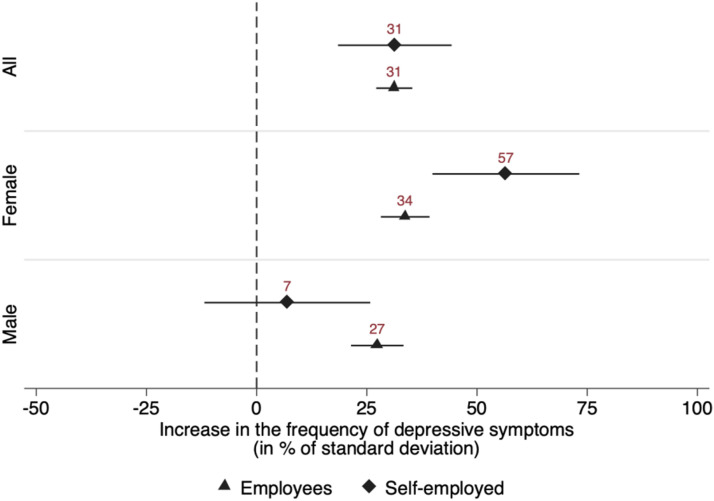
Mental health changes among the self-employed and employed people. *Note:* Figure 6 displays the linear combinations of coefficients for the respective groups in Table A4. 95% confidence intervals are shown in brackets and are represented by the horizontal whiskers in the figure.

We start by investigating whether the self-employed, on average, experienced a stronger decline in mental health than employees due to the pandemic, and whether there were differential effects by the incidence of income losses. In doing so, we also examine gender differences in mental health changes. Table A4 shows our estimates of *β*_1_ from Equation (1), in which we distinguish between the self-employed and employees. Column (1) displays results for the whole sample, while column (2) and column (3) show the estimates of *β*_1_ for women and men, respectively. All estimates control for individual and year fixed effects. The estimates in the first row correspond to the change in the frequency of depressive symptoms between 2019 and 2020 for the baseline group, that is, employees, while the estimates in the second row show the *differential* effect for the self-employed. This differential effect represents the estimated causal effect of the pandemic on the self-employed. The overall change for the self-employed is then represented by the linear combination of both coefficients, that is, *β*_1_+*β*_2_, which is shown in Figure 6. For ease of interpretation, we base our discussion mostly on these figures, which represent the overall changes for the respective groups.

As Figure 6 and the corresponding estimates in column (1) of Table A4 reveal, the frequency of depressive symptoms increased significantly between 2019 and 2020 for both employees and self-employed individuals. At first glance, there appear to be no significant differential changes. For employees, the frequency of depressive symptoms, as measured by the PHQ-4 score, increased by about 31% of a standard deviation. For the self-employed, the corresponding change is, contrary to our hypothesis, identical, pointing toward the absence of an effect of self-employment compared to organizational employment. However, this masks substantial heterogeneity by gender. The frequency of depressive symptoms in self-employed women increased by about 57% of a standard deviation—considerably more than in female employees. Thus, the difference of 23% of a standard deviation between the two groups is the estimated effect of the pandemic on self-employed women relative to female employees. We judge this effect to be of small to medium size (Cohen, 2013). For men, the reverse is true: whereas male employees experienced similar levels of mental strain to female employees, the change in the PHQ-4 score for self-employed men is indistinguishable from zero.

These findings offer partial support for hypothesis *H1* in that self-employed women experienced a considerably greater increase in the frequency of depressive symptoms than both male and female employees. However, the hypothesis does not hold true for men: self-employed men experienced relatively small, if any, changes in mental health. Moreover, our findings show that the negative changes in mental health among the self-employed were heavily concentrated among women.

**Figure 7.**
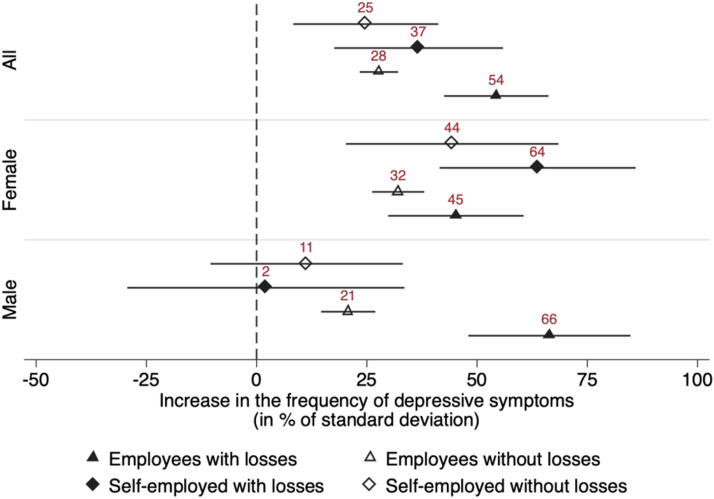
Mental health changes of the self-employed and employees with and without income losses. *Note:* Figure 7 displays the linear combinations of coefficients for the respective groups in Table A5. 95% confidence intervals are shown in brackets and are represented by the horizontal whiskers in the figure.

To investigate the role of income losses, that is, hypothesis *H1a,* we re-estimate Equation (1), distinguishing employees and self-employed individuals by whether they experienced income losses due to the COVID-19 pandemic. Again, we show results for the whole sample and separately by gender, which are visualized in Figure 7. The corresponding estimates are given in Table A5.^15^

Regardless of the impact of the COVID-19 pandemic on individual incomes, all four groups experienced a strong increase in the frequency of depressive symptoms. Moreover, the results reveal significant heterogeneities within the groups of employees and self-employed individuals. The estimates suggest that the decline in mental health was, in general, greater for those who suffered financially from the crisis. As shown in the upper part of Figure 7, the PHQ-4 score of employees who suffered income losses increases by about 54% of a standard deviation. This change is statistically significant. The change in mental health of self-employed individuals with income losses is about 37% of a standard deviation, yet does not differ from that of their employed counterparts in a statistical sense. Importantly, for both employment groups, the point estimate for those who did not suffer any losses is notably smaller.

Yet again, this masks substantial heterogeneity by gender. In fact, the observed decrease in mental health of the self-employed appears to be driven entirely by women. Self-employed women who did not suffer financial losses experienced a similar magnitude of increases in mental distress to employed women who did suffer financial losses. Self-employed women who incurred financial losses showed the largest increase in the PHQ-4 score, amounting to almost 64% of a standard deviation. While the point estimates suggest a difference of around 19% of a standard deviation between self-employed women who experienced income losses and organizationally employed women who experienced income losses, this difference is imprecisely estimated such that we cannot rule out the absence of differential mental health effects from a statistical perspective. The difference between self-employed women who experienced income losses and self-employed women who did not experience income losses is of similar magnitude. Conversely, for self-employed men, the change in mental health is indistinguishable from zero in economic and statistical terms, regardless of whether they incurred financial losses. The decline in mental health among men seems to be largely driven by male employees with income losses, despite the fact that short-time work often reduced the magnitude of losses among employees. In the section *Income and Mental Health*, we discuss how income and income uncertainty can negatively affect mental health. One possible explanation for this finding may be that the self-employed are more accustomed to fluctuations in income (Baron, 2008). However, this is not corroborated by our findings for women.

Overall, our findings provide partial support for hypothesis *H1a* in that income losses play a significant role in explaining differential changes in mental health, both between and within the groups of self-employed individuals and employees. While the incidence of income losses is significantly larger among the self-employed (Table A3), both self-employed and organizationally employed people experienced negative changes in mental health when they incurred income losses. In particular, the observed decline in mental health was greatest for self-employed women and organizationally employed men. Self-employed men are the exception, with no statistically significant changes in their PHQ-4 score, regardless of whether they experienced income losses.

Our findings in this section also hold if we compare the self-employed to employees in managerial positions only, a group that one may argue is more directly comparable to the self-employed than the entire population of employees. The corresponding robustness check is displayed in Appendix B. Compared to the regression with all employees as the control group, the corresponding differences are smaller. This aligns well with our expectations. Importantly, the results of this exercise leave our substantive conclusions unchanged.

### Procedural Utility and Resilience

**Figure 8.**
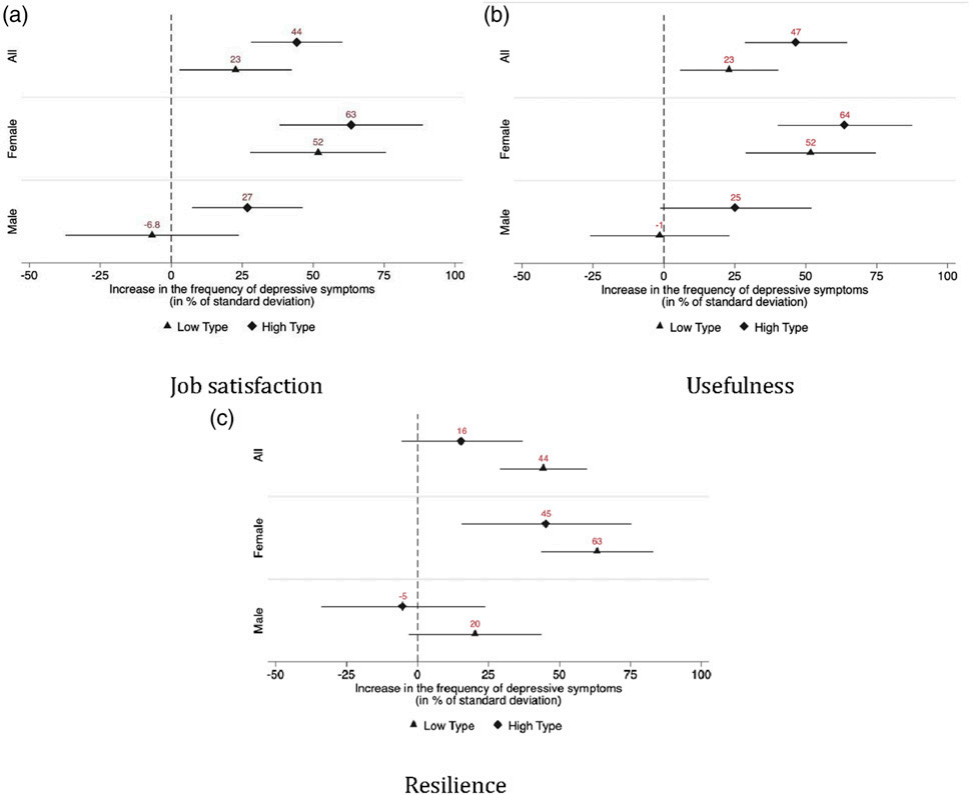
Mental health changes of the self-employed, by levels of procedural utility and resilience. *Note:* Figure 8 displays the linear combinations of coefficients for the respective groups in Table A6. 95% confidence intervals are shown in brackets and are represented by the horizontal whiskers in the figure.

Having isolated the effect of the pandemic on the mental health of the self-employed, we shift our focus to examining differential mental health changes among them to identify potential drivers of the observed increase in the PHQ-4 score of the self-employed. We drop employees from our sample and perform intra-group comparisons among the self-employed, both for the entire sample of self-employed individuals and separately by gender. We start by replacing *Selfemp*_*i*_ with the respective variable under consideration and compare the change in the frequency of depressive symptoms between 2019 and 2020 for those self-employed individuals who are below or at the median of the respective variable (low type) to those above the median (high type). The results are shown in Figure 8 and the corresponding Table A6.

*Procedural Utility:*Figure 8(a)/8(b) and Panel A/B of Table A6 provide evidence that self-employed individuals who derive larger procedural utility from their work experienced larger decreases in mental health. For both “job satisfaction” and “usefulness,” those who score above the median experienced an increase in the PHQ-4 score that is roughly twice as large as for those who score at or below the median. A separate analysis by gender shows no notable between-group differences among self-employed women. However, the results indicate that, among self-employed men, those who are more satisfied with their job and those who consider their work more useful, displayed an increase in their PHQ-4 score, in contrast to the “low types”.

*Resilience:*Figure 8(c) and Panel C of Table A6 provide support for our hypothesis that resilience is a protective factor for mental health. Individuals who are very resilient, that is, who score above the median on our resilience measure, experienced relatively little, if any, change in the frequency of depressive symptoms between 2019 and 2020. By contrast, the PHQ-4 scores of less resilient self-employed individuals increased by about 44% of a standard deviation on average. The separate analysis by gender adds little to explaining the observed gender differences in mental health changes: self-employed women suffered from significant increases in their PHQ-4 score regardless of how resilient they are. For resilient self-employed men, the change in mental health is statistically not different from zero, while less resilient men experienced an increase in their PHQ-4 score by about 20% of a standard deviation.

Overall, these results provide support for hypotheses *H2a* and *H2b*. Self-employed individuals with higher job satisfaction and those with higher reported levels of usefulness, our measures of procedural utility, experienced larger increases in the frequency of depressive symptoms. This is particularly the case for men. By contrast, resilience protected self-employed individuals from sizable increases in the PHQ-4 score during the COVID-19 pandemic, lending support to hypothesis *H3*. Still, this is less the case for self-employed women, as they experienced significantly more negative changes in mental health even if they score high on our resilience measure.

### Structural Context and Mechanisms

In the *Main Results* section, we established that self-employed women experienced the sharpest increase in the frequency of depressive symptoms, whereas we observe hardly any changes in the PHQ-4 score of self-employed men on average. In the following, we explore potential reasons underlying this disproportionate change in the frequency of anxiety and depressive symptoms among self-employed women. We investigate whether self-employed women were affected differently than self-employed men by government restrictions affecting business operations and whether being affected by such restrictions translates into a decline in mental health. We also test whether having children or, more directly, whether the availability of help with childcare acts as a driver of the differential mental health changes among self-employed women during the pandemic.

**Figure 9.**
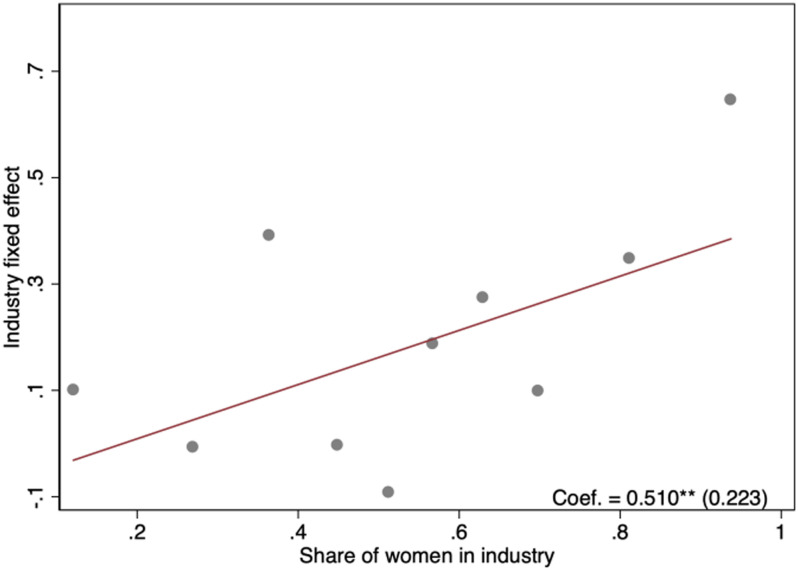
Share of women in industries affected by restrictions. *Note:* Figure 9 displays the association between the probability of facing restrictions and the share of women in the respective industry. We proceed in two steps: First, we estimate a regression of a binary indicator whether a self-employed individual was affected by government restrictions related to the COVID-19 pandemic on the NACE Rev. 2 industry dummies. We then plot the obtained industry coefficients against the share of women in the respective industry in our working sample. The figure corresponds to a binned scatterplot. The regression coefficients stem from an OLS regression of the industry fixed effects on the share of women in the respective industries. Robust standard errors are in parentheses and read * *p<*0.10, ** *p<*0.05, *** *p<*0.01.

*Government-imposed restrictions on businesses:* In the SOEP-CoV questionnaire, self-employed respondents were asked whether their business was directly affected by pandemic restrictions such as limitations on opening hours. 61% of those who were directly affected by government-imposed restrictions are women. This strongly correlates with industry affiliation. In Figure 9, we plot the industry coefficients of a regression of the binary indicator of whether a self-employed individual was affected by government restrictions on industry dummies against the share of women in the respective industry.^16^ Thus, the industry-specific probability of being affected by government restrictions is positively associated with the share of women in the respective industry.

**Figure 10.**
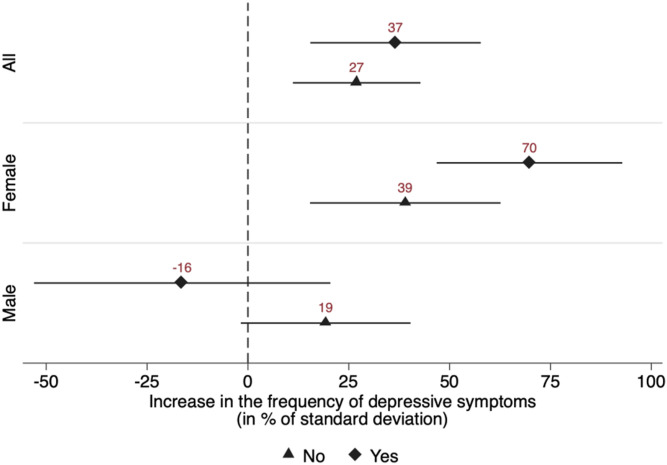
Mental health changes of the self-employed, by whether they were affected by restrictions. *Note:* Figure 10 displays the linear combinations of coefficients for the respective groups in Table A7. 95% confidence intervals are shown in brackets and are represented by the horizontal whiskers in the figure.

We use this indicator to assess whether self-employed individuals facing such restrictions experienced differential changes in their mental health when compared to those who were unaffected by government measures. The results are displayed in Figure 10 and Panel A of Table A7. Thus, we compare the change in mental health between 2019 and 2020 for those unaffected by restrictions to the PHQ-4 score of those who were affected.

The results show that the increase in the frequency of depressive symptoms of self-employed women directly affected by government-imposed restrictions was 79% larger than for those unaffected and that this difference is statistically significant at the 10% level (Table A7). For self-employed men, on the other hand, there appears to be no such effect. Together, these findings support hypothesis *H4*, providing evidence that women not only sort disproportionately into industries that have been more strongly affected by the pandemic, but that this translated into a decrease in mental health among self-employed women during the pandemic.^17^

*Closure of schools and childcare facilities:* To investigate the impact of increased childcare needs on mental health, we start by testing whether self-employed individuals who have children of school age or younger living in the household experienced a differential change in mental health compared to those without children in the household. However, the presence of children alone gives us only limited information on how much an individual respondent’s childcare activities changed. For instance, individuals may have private help with childcare from friends or relatives. To control for such factors, we use a simple proxy in a second step. Respondents were further asked to indicate how many hours of childcare were provided by individuals other than themselves. This includes both childcare by private individuals as well as by schools and daycare centers. From this, we construct a proxy that is zero if the individual had no children living in the household or if the individual had help with childcare for at least a few hours a week. The proxy is equal to one if the individual had children and no help with childcare.

**Figure 11.**
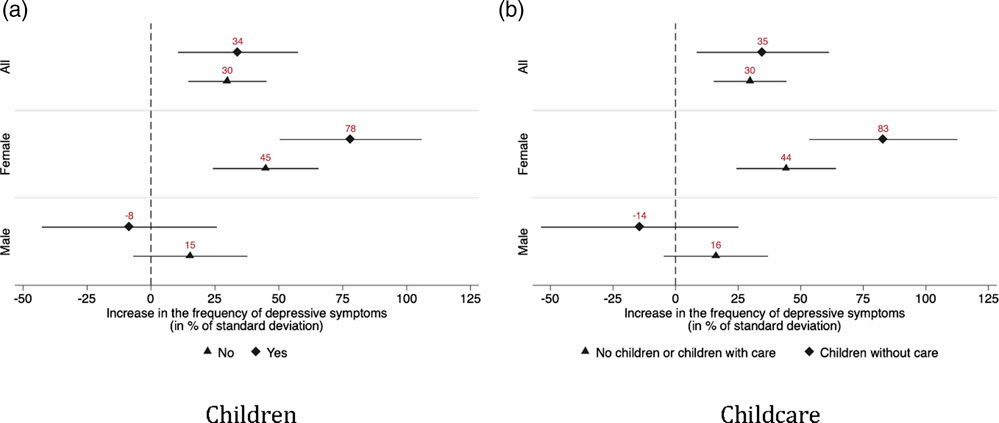
Mental health changes of the self-employed by whether they had children in the household and whether they had help with childcare. *Note:* Figure 11 displays the linear combinations of coefficients for the respective groups in Table A8. 95% confidence intervals are shown in brackets and are represented by the horizontal whiskers in the figure.

The results in Figure 11(a) and Panel A of Table A8 reveal that having children increased the frequency of depressive symptoms among self-employed women during this pandemic. For men, the presence of a child had no differential impact and, if anything, went in the opposite direction. When we use our proxy for childcare, the results are qualitatively similar. As shown in Figure 11(b) and Panel B of Table A8, self-employed women with children and no help with childcare experienced an increase in the PHQ-4 score that is almost twice as great as the increase for self-employed women who have no children or who have children but at least some help with childcare. For self-employed men, however, we find no such effect. These results lend support to hypothesis *H5*.

### Long-Term Effects

Finally, we investigate whether the estimated mental health effects persisted over a longer period. To this end, we use the second wave of data from SOEP-CoV, which was collected in January and February 2021. We re-estimate Equation (1), but instead of comparing the years 2019 and 2020, we use the years 2019 and 2021. As before, we anchor the employment status in 2020, thus comparing individuals who were self-employed in 2020 to employees. The advantage of this approach is that we follow the same set of individuals over time such that our estimate is not confounded by labor market transitions between years.^18^ The results are shown in Table A9. As before, there is no significant difference between the self-employed and employees, with women experiencing stronger increases in their PHQ-4 scores than men. However, the within-gender differences between organizationally employed and self-employed women seem to vanish over time. Importantly, the effects of the COVID-19 pandemic on mental health appear persistent and are only slightly smaller in magnitude compared to 2020. While the pandemic is still ongoing at the time of writing, these findings suggest that its detrimental effects on mental health go beyond a mere short-run setback, with corresponding implications for policymakers.

## Discussion and Conclusion

We analyze how the shock associated with the COVID-19 pandemic, comprising various policy measures aimed at containing the spread of the virus, affected the mental health of the self-employed. Using a difference-in-differences approach, we compare mental health changes in the self-employed with mental health changes in employees between 2019 and 2020 to provide a first causal estimate of the effects of the pandemic on the mental health of self-employed individuals. Exploiting the richness of our data, we can identify several factors that drive the mental health responses of self-employed individuals, focusing in particular on differences by gender. Finally, we provide initial evidence on the extent to which the observed effects have persisted through the pandemic so far.

Our analysis provides several key insights: first, self-employed people and employees showed similar and substantial declines in mental health in the first year of the pandemic. This indicates that in terms of mental health the self-employed were, on average, not more strongly affected by the pandemic than employees. However, this masks substantial heterogeneity by gender, leading to our second insight: the mental health of self-employed women in particular has deteriorated since the start of the pandemic. Third, income losses due to the pandemic are associated with a worsening of mental health in the affected individuals. Fourth, resilience helped the self-employed cope with the negative impact of the pandemic on mental health. Fifth, our results indicate that the deterioration in mental health persisted into early 2021.

Further differentiating by gender, our results shed light on potential reasons underlying the gendered impact of the pandemic on the self-employed. Self-employed women experienced the largest increases in the frequency of anxiety and depressive symptoms during the COVID-19 pandemic, especially those who incurred financial losses resulting from pandemic restrictions. Moreover, self-employed women directly affected by restrictions on their business operations faced a worsening of their mental health. Likewise, the closure of schools and daycare centers imposed an additional burden on self-employed women with children, who had to reconcile increased childcare needs with the responsibilities of running a business. Thus, it appears that the differential impact of restrictions on doing business, aimed at containing the spread of SARS-CoV-2, helps explain the increases in the frequency of depressive symptoms in self-employed women. This is not only because of the effect these policy measures had on income, but also due to their negative impact on job control.

The pandemic affected self-employed men very differently. Their mental health was largely unaffected by the crisis and appeared to be robust against monetary losses. This stands in stark contrast to the strong decline in mental health among organizationally employed men who experienced income losses. It thus appears that the restrictions of entrepreneurial freedom brought about by the pandemic weigh heavier on the mental health of self-employed men than the possible financial losses associated with these restrictions. This reinforces the notion that non-pecuniary aspects constitute an important part of the returns the self-employed accrue from their activities. We do find some evidence of increases in the frequency of anxiety and depressive symptoms among self-employed men who lack resilience and those who derive more procedural utility from their work. Overall, our results imply that self-employed men are the only group (among those we analyze here) who made it through the pandemic without much deterioration in their mental health. However, the finding that male employees who incurred income losses from the pandemic did experience a significant worsening in mental health implies that the deterioration in mental health is not simply a gender issue. One possible explanation for this finding could be favorable sorting into self-employment (Rietveld et al., 2015; Stephan et al., 2020), or that self-employed men are more used to and better equipped to deal with variations in income (Baron, 2008).

Our findings have implications for the subsequent development of entrepreneurship. During the COVID-19 pandemic the self-employed have faced financial losses that for many of them have led to an existential threat. In such a crisis, being able to make the right decision becomes crucial. However, if self-employed individuals simultaneously experience a negative income shock and a worsening in mental health, which we find is especially frequent in women, this can negatively influence their decision-making processes and productivity (e.g., Hessels et al., 2018). Negative mental health shocks are indeed associated with lower annual incomes from self-employment (Hatak & Zhou, 2021). One would expect the COVID-19 pandemic to have negative second-order effects on the performance of the self-employed if mental distress increases in its wake. Such effects could be amplified through a downward spiral of depression and negative economic outcomes (Gorgievski et al., 2010; Ridley et al., 2020), which would not just increase private costs, but also impose societal costs in terms of job losses, higher health care costs, and lower social welfare. Hessels et al. (2018), for instance, show that an increase in depressive symptoms of one unit, on a scale from 0 to 10, is associated with an increase of the likelihood of exiting self-employment by about 1.7 percentage points, or 10%. The COVID-19 pandemic may therefore have negative implications for the long-term entrepreneurial survival of affected individuals.^19^

Overall, we conclude that particularly self-employed women are vulnerable in this pandemic crisis. The observed negative impact of the COVID-19 pandemic on their mental health may also result in a higher reluctance of women to become self-employed. Self-employment would, furthermore, be considerably less attractive in industries that were particularly affected by the pandemic, and these are industries that are dominated by women. This should also be seen in the context of the slowly increasing willingness of women to enter self-employment (Fritsch et al., 2015). If women exit self-employment disproportionately often as a consequence of the pandemic, the gender gap in self-employment may widen again.

Our results have important policy implications. Employees are typically insured against labor market risks through the social security system. However, in many countries, including Germany, the social security system is not well equipped to smooth income from self-employment during phases of economic hardship. Policymakers have limited latitude in implementing non-pharmaceutical interventions to contain the spread of the virus, but they could do much to build confidence in the economy by providing the self-employed with steady and reliable support that enables them to weather financial losses caused by a systemic shock for which they bear no responsibility. This could mitigate the mental strain on the self-employed and increase the likelihood of business survival. In addition, policymakers should consider implementing publicly funded direct prevention measures such as support networks, coaches, or telephone hotlines that help in addressing symptoms of depression and anxiety at an early stage. This could also help people build psychological resilience, which, according to our results, moderates the relationship between the COVID-19 pandemic and mental distress. This is certainly a long-term task since traits like resilience are likely more mutable during adolescence. However, the (non-)monetary returns are potentially large since societies will undoubtedly have to weather further crises in the future, caused by economic downturns, pandemics, or climate change.

## Figures and Tables

**Figure A1.**
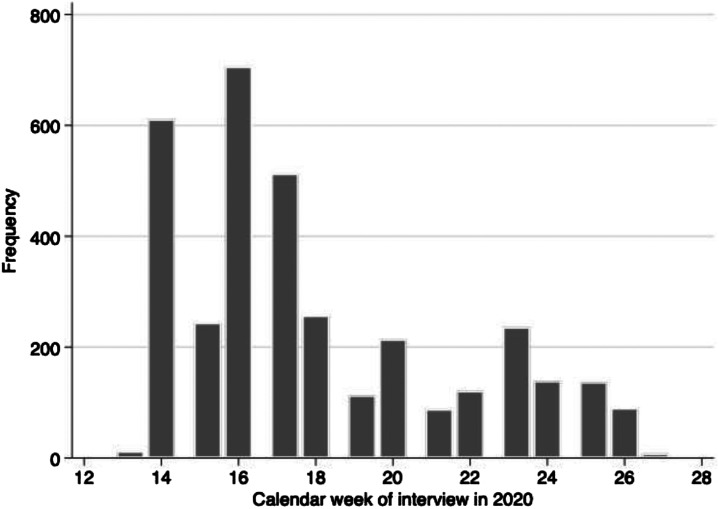
Distribution of interviews over calendar weeks in 2020

**Figure A3.**
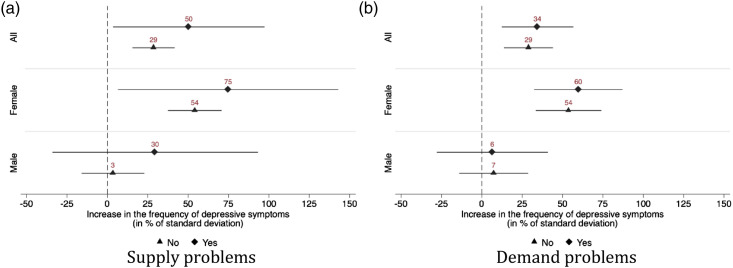
Linear combination of coefficients, Panel B and C of Table A7. (a) Supply problems and (b) demand problems. *Note:* Figure A3 displays the linear combinations of coefficients for the respective groups in Table A7. 95% confidence intervals are shown in brackets and are represented by the horizontal whiskers in the figure.

**Figure B4.**
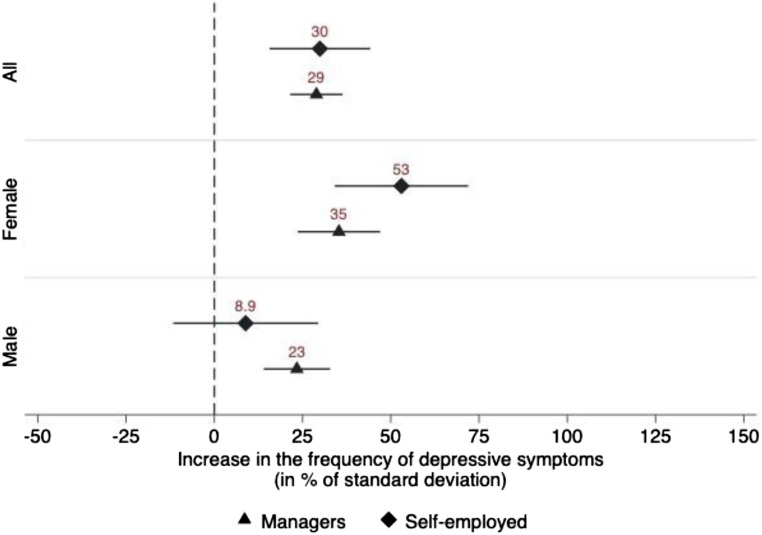
Mental health changes of the self-employed and employed managers. *Note:* Figure B4 displays the linear combinations of coefficients for the respective groups in Table B10. 95% confidence intervals are shown in brackets and are represented by the horizontal whiskers in the figure.

**Figure B5.**
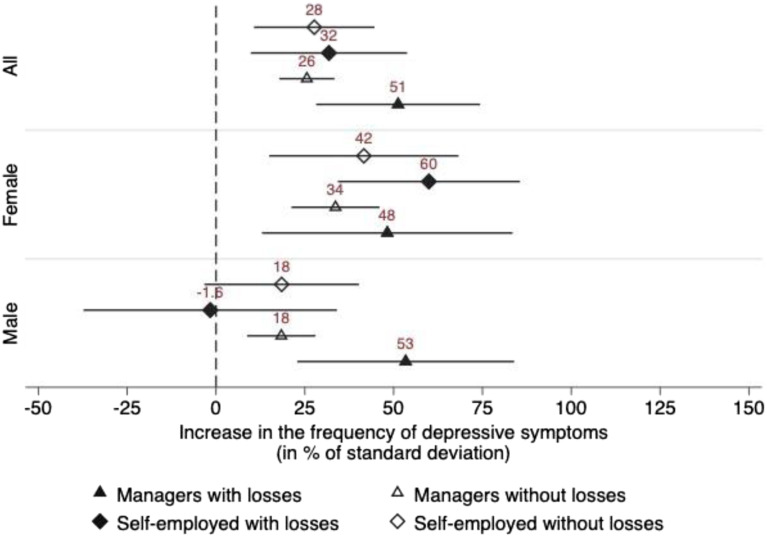
Mental health changes of the self-employed and employed managers with and without income losses. *Note:* Figure B.5 displays the linear combinations of coefficients for the respective groups in Table B11. 95% confidence intervals are shown in brackets and are represented by the horizontal whiskers in the figure.

## Notes

1. Nikolova (2019) is a notable exception.

2. *Kurzarbeit* is a short-time work compensation scheme in Germany in which the Federal Employment Agency (FEA) covers up to 67% of the net income of employees whose working time has been temporarily reduced. The maximum amount is paid out if working hours are reduced to zero.

3. Only 207,409 out of four million self-employed individuals, or 5%, had unemployment insurance in 2012 (Evers et al., 2013).

4. Individuals in the BHPS are asked how satisfied they are with “being able to use their own initiative” and “the actual work itself,” both reflecting procedural aspects of work.

5. See also the discussion on job control in the context of the JDC model in *The JDC Model*.

6. That is, if individuals who derive high procedural utility in normal times are suddenly unable to work, they lose part of their endowed procedural utility. Evaluated from a reference point, the utility function is flatter in the gain domain than in the loss domain, and losses therefore weigh heavier than gains. This is typically referred to as loss aversion (Tversky & Kahneman, 1991).

7. Anwar et al. (2023) measure resilience on the individual level but argue that higher individual-level resilience extrapolates to the organizational level.

8. According to the OECD (2017), 9 out of 10 self-employed women in Germany work in the service sector. This is true for only about two thirds of men.

9. The SOEP-CoV survey is part of the project “The Spread of the Coronavirus in Germany: SocioEconomic Factors and Consequences,” funded by the German Federal Ministry of Education and Research.

10. We used SOEPv35. DOI: 10.5684/soep-core.v35. We also used preliminary SOEP data for 2019.

11. We consider self-employment an occupational status that encompasses individuals working on their own account and risk. An alternative, narrower definition concentrating on entrepreneurs would emphasize the role of seeking novelty and opportunity (Gorgievski et al., 2010). In the context of mental health questions, Gorgievski and Stephan (2016) explain on page 439: “*Other aspects of entrepreneurship, such as the agency and uncertainty it involves, felt responsibility, and decision-making freedom appear more relevant than novelty from the perspective of psychology, which, as a discipline, is focused on understanding intra- and inter-individual processes of action*.” For this reason and in the sense of an occupational status, we mostly use the term self-employment, but introduce the term entrepreneurship where it seems appropriate.

12. The distribution of observations of our final sample over calendar weeks in 2020 is shown in Figure A1.

13. We chose this dummy variable because we are interested in the effect of the incidence of income losses on mental health and not on the effect of the magnitude of those losses on mental health. Moreover, note that the SOEP-CoV questionnaire does not allow inference of pre-pandemic income levels, making the information on the magnitude of changes less informative.

14. This figure is derived as follows: 55% = [62.9%*151+47.4%*156]/307.

15. Here, the baseline group consists of employees with income losses. The first row of Table A5 therefore shows the change in the frequency of depressive symptoms for employees who stated that their income had fallen as a direct consequence of the COVID-19 crisis. The second to fourth rows show the differential change of employees without income losses, the self-employed with income losses, and the self-employed without income losses, respectively. These estimates should be interpreted relative to the baseline group.

16. The SOEP-CoV questionnaire further asked self-employed respondents whether the COVID-19 pandemic led to supply shortages for their business and whether they faced demand problems due to the pandemic. In Figure A3(a) and (b) as well as in Panel B and Panel C of Table A7, we repeat this analysis, but use indicators for supply shortages and demand problems instead. We find no evidence of a relationship with mental health.

17. The SOEP-CoV questionnaire further asked self-employed respondents whether the COVID-19 pandemic led to supply shortages for their business and whether they faced demand problems due to the pandemic. In Figure A3(a) and (b) as well as in Panel B and Panel C of Table A.7, we repeat this analysis, but use indicators for supply shortages and demand problems instead. We find no evidence of a relationship with mental health.

18. Note, however, that we lost some observations due to attrition between the two SOEP-CoV waves.

19. In this context, it should be kept in mind that episodes of depression are shown to shorten patients’ life expectancy. In their meta-analysis, Cuijpers et al. (2014) report that depression increases mortality by about 50%.

20. Note that the loss of significance likely results from a loss in degrees of freedom due to the smaller sample size, leading to an increase in statistical uncertainty.

## Appendix A

**Table A1.** Variable Descriptions

(1)	(2)	(3)
Variable	Description	Year of Origin
PHQ4-score	Sum score of four items used to infer the frequency of symptoms of anxiety and depression, range 0–12.	2019–2020
Income decrease (earnings)	Indicator reflecting decrease of monthly gross income decrease due to COVID-19 pandemic.	2020
Female	Indicator for being female.	pre-2020
Age	Difference between survey year and birth year.	pre-2020
Migration background	Indicator for having direct or indirect migration background.	pre-2020
Resilience	Question on how quickly individuals tend to recover following difficult times, 5-point scale.	2020
Usefulness	Question on how whether individuals consider their activity valuable and useful in life, 11-point Likert scale.	2019
Household size	Number of household members.	2019
Household net income	Monthly household net income.	2019
Married	Indicator for being married.	2019
Children (school age or younger)	Indicator reflecting the presence of a child of school age or younger living in the household.	2020
Childcare hours	Number of hours spent on childcare on a usual weekday.	2020
Childcare proxy	Indicator that is 1 if children of school age or younger are living in the household and no help with childcare is available.	2020
Basic school leaving degree	Indicator for categories 0 “in school” to 1c “basic vocational education” according to the Comparative Analysis of Social Mobility in Industrial Nations (CASMIN)-scale.	Last available information in 7 year window pre-2020
Intermediate school leaving degree	Indicator for categories 2b “intermediate general qualification” to 2c voc “vocational maturity certificate” according to the CASMIN-scale.	Last available information in 7 year window pre-2020
Tertiary school leaving degree	Indicator for categories 3a “lower tertiary education” or 3b “higher tertiary education” according to the CASMIN-scale.	Last available information in 7 year window pre-2020
Unemployment experience	Generated unemployment experience from “pgen.dta” of the SOEP v.35.	2018
NACE 2 code	Two-digit NACE Industry—Sector. Missing values, e.g., due to unemployment in 2019, are coded as separate category.	2019
Subject to regulation	Indicator reflecting whether self-employed individuals’ business was subject to regulations aimed at containing COVID-19, e.g., regulation of opening hours.	2020
Supply problems	Indicator reflecting whether self-employed individuals’ business suffered from shortages of intermediate goods.	2020
Demand problems	Indicator reflecting whether the self-employed individuals’ business suffered from cancellation of their services and goods, i.e., demand shortage.	2020

*Note:* Table A1 provides information on variables and their year of origin.

**Table A2.** Summary Statistics

	(1)	(2)	(3)	(4)
	Self-Employed		Employees	
	Income Losses	No Income Losses	Income Losses	No Income Losses
PHQ4-score	2.367	1.623	2.506	2.114
	(2.126)	(1.644)	(2.204)	(1.897)
Income decrease (earnings)	1.000	0.000	1.000	0.000
Demographics:				
Female	0.562	0.406	0.568	0.614
Age	52.124	55.268	46.969	47.060
	(10.655)	(11.623)	(10.419)	(10.429)
Migration background	0.189	0.116	0.269	0.189
Factors governing mental health:				
Resilience	4.219	4.181	3.954	4.041
Job satisfaction (2019)	7.550	8*.*107	7.136	7.285
Usefulness (2019)	7.822	7.964	7.475	7.658
Household context:				
Household size (2019)	2.568	2.638	2.894	2.802
	(1.409)	(1.439)	(1.344)	(1.382)
Household net income (2019)	4390.155	4860.599	3594.680	3910.684
	(5146.510)	(3503.424)	(1665.930)	(2003.033)
Married (2019)	0.574	0.674	0.614	0.583
Children (school age or younger)	0.379	0.319	0.496	0.464
Child-care hours	2.560	1.529	3.048	2.273
Child-care proxy	0.337	0.275	0.415	0.374
Education (ref. basic:)				
Intermediate	0.386	0.353	0.563	0.474
Tertiary	0.530	0.529	0.261	0.374
Unemployment experience	0.582	1.025	1.244	0.778
Revenue-reducing events in the wake of COVID-19:				
Subject to regulation	0.598	0.283		
Supply problems	0.142	0.109		
Demand problems	0.663	0.167		
Number of individuals	169	138	417	2765

*Note:* Table A2 displays mean and standard deviations, in parentheses, for self-employed individuals and employees in 2020. The sample is restricted on observing all variables used in estimation. For some of the other variables the number of observations may be smaller.

**Table A3.** Summary Statistics by Gender

	(1)	(2)	(3)	(4)
	Self-Employed		Employees	
	Female	Male	Female	Male
PHQ4-score	2.444	1.635	2.336	1.900
	(2.080)	(1.745)	(1.999)	(1.825)
Income decrease (earnings)	0.629	0.474	0.122	0.144
Demographics:				
Female	1.000	0.000	1.000	0.000
Age	51.881	55.141	47.048	47.048
	(10.378)	(11.739)	(10.054)	(10.983)
Migration background	0.146	0.167	0.193	0.210
Factors governing mental health:				
Resilience	4.086	4.314	3.991	4.088
Job satisfaction (2019)	7.843	7.754	7.269	7.260
Usefulness (2019)	7.927	7.846	7.677	7.567
Household context:				
Household size (2019)	2.596	2.603	2.862	2.740
	(1.372)	(1.471)	(1.341)	(1.429)
Household net income (2019)	4279.163	4917.130	3789.717	3993.071
	(5009.165)	(3886.933)	(1936.784)	(2002.271)
Married (2019)	0.596	0.641	0.577	0.603
Children (school age or younger)	0.351	0.353	0.489	0.436
Child-care hours	2.616	1.587	2.863	1.616
Child-care proxy	0.318	0.301	0.397	0.351
Education (ref. basic:)				
Intermediate	0.409	0.333	0.529	0.418
Tertiary	0.503	0.556	0.336	0.395
Unemployment experience	0.888	0.678	0.946	0.675
Revenue-reducing events in the wake of COVID-19:				
Subject to regulation	0.570	0.346		
Supply problems	0.119	0.135		
Demand problems	0.470	0.410		
Number of individuals	151	156	1936	1246

*Note:* Table A3 displays mean and standard deviations, in parentheses, for self-employed individuals and employees in 2020. The sample is restricted on observing all variables used in estimation. For some of the other variables the number of observations may be smaller.

**Table A4.** Differences in Mental Health Between Self-Employed Individuals and Employees

	(1)	(2)	(3)
	All	Female	Male
Baseline:			
Employees	0.312***	0.337***	0.274***
	(0.021)	(0.028)	(0.030)
Differential effect:			
Self-employed	0.001	0.228**	−0.204**
	(0.069)	(0.090)	(0.101)
Observations	6978	4174	2804
R2	0.67	0.66	0.67

*Note:* Table A4 displays the effect of being in different employment states on mental health during the COVID-19 pandemic. Column (1) to (3) each correspond to a regression of the PHQ-4 score on year indicators, individual fixed effects and interactions with the group indicator and the indicator for the survey year 2020. The PHQ-4 score has been standardized to have mean zero and standard deviation one. Standard errors are clustered on the individual level and in parentheses. **p<*0.10, ***p<*0.05, ****p<*0.01.

**Table A5.** Differences in Mental Health Between Different Groups

	(1)	(2)	(3)
	All	Female	Male
Baseline: (1) Employees with income loss	0.544***	0.452***	0.664***
	(0.060)	(0.078)	(0.094)
Differential effect: (2) Employees without income loss	−0.266***	−0.131	−0.456***
	(0.064)	(0.084)	(0.099)
(3) Self-employed with income loss	−0.176	0.185	−0.643***
	(0.115)	(0.138)	(0.186)
(4) Self-employed without income loss	−0.296***	−0.008	−0.551***
	(0.103)	(0.146)	(0.146)
*p*-value (3)–(4)	0.352	0.247	0.635
Observations	6978	4174	2804
R2	0.67	0.66	0.68

*Note:* Table A5 displays the effect of being in different employment states on mental health during the COVID-19 pandemic. Column (1) to (3) each correspond to a regression of the PHQ-4 score on year indicators, individual fixed effects and interactions with the group indicator and the indicator for the survey year 2020, respectively. The PHQ-4 score has been standardized to have mean zero and standard deviation one. Standard errors are clustered on the individual level and in parentheses. * *p <* 0.10, ** *p <* 0.05, *** *p <* 0.01.

**Table A6.** Procedural Utility and Resilience

A: Job Satisfaction			
	(1)	(2)	(3)
	All	Female	Male
Baseline:			
Low type	0.227**	0.517***	−0.068
	(0.101)	(0.122)	(0.156)
Differential effect:			
High type	0.216*	0.116	0.336*
	(0.130)	(0.177)	(0.185)
Observations	544	268	276
*R*2	0.69	0.75	0.66
B: Usefulness		
Baseline:			
Low type	0.230***	0.517***	−0.015
	(0.088)	(0.117)	(0.125)
Differential effect:			
High type	0.235*	0.121	0.268
	(0.128)	(0.168)	(0.185)
Observations	614	302	312
*R*2	0.69	0.75	0.66
C: Resilience		
Baseline:			
Low type	0.444***	0.633***	0.203*
	(0.079)	(0.101)	(0.120)
Differential effect:			
High type	−0.287**	−0.179	−0.253
	(0.134)	(0.183)	(0.190)
Observations	614	302	312
*R*2	0.70	0.76	0.66

*Note:* Panel A of Table A6 displays the effect of job satisfaction on mental health during the COVID-19 pandemic. Panel B displays the effect of usefulness on mental health during the COVID-19 pandemic. Panel C displays the effect of resilience on mental health during the COVID-19 pandemic. Low (high) types are those who score below (above) the median on the respective variable. Column (1) to (3) each correspond to a regression of the PHQ-4 score on year indicators, individual fixed effects and interactions with the group indicator and the indicator for the survey year 2020. The PHQ-4 score has been standardized to have mean zero and standard deviation one. Standard errors are clustered on the individual level and in parentheses. * *p <* 0.10, ** *p <* 0.05, *** *p <* 0.01.

**Table A7.** COVID-19-related events that affect the self-employed's business activities

A: Restrictions			
	(1)	(2)	(3)
	All	Female	Male
Baseline:			
No	0.270***	0.390***	0.193*
	(0.081)	(0.120)	(0.107)
Differential effect:			
Yes	0.096	0.308*	−0.356
	(0.135)	(0.168)	(0.216)
Observations	614	302	312
*R*2	0.69	0.76	0.66
B: Supply		
Baseline:			
No	0.286***	0.541***	0.034
	(0.067)	(0.085)	(0.099)
Differential effect:			
Yes	0.218	0.207	0.261
	(0.248)	(0.358)	(0.339)
Observations	614	302	312
*R*2	0.69	0.75	0.65
C: Demand		
Baseline:			
No	0.289***	0.537***	0.073
	(0.077)	(0.104)	(0.109)
Differential effect:	0.056	0.061	−0.008
Yes	(0.137)	(0.174)	(0.207)
Observations	614	302	312
*R*2	0.69	0.75	0.65

*Note:* Panel A of Table A7 displays the effect of being affected by government restrictions on mental health during the COVID-19 pandemic. Panel B displays the effect of being affected by supply shortages on mental health during the COVID-19 pandemic. Panel C displays the effect of being affected by demand shortages on mental health during the COVID-19 pandemic. Column (1) to (3) each correspond to a regression of the PHQ-4 score on year indicators, individual fixed effects and interactions with the group indicator and the indicator for the survey year 2020. The PHQ-4 score has been standardized to have mean zero and standard deviation one. Standard errors are clustered on the individual level and in parentheses. * *p<*0.10, ** *p<*0.05, *** *p<*0.01.

**Table A8.** Children

A: Children			
	(1)	(2)	(3)
	All	Female	Male
Baseline:			
No	0.299***	0.449***	0.154
	(0.078)	(0.105)	(0.114)
Differential effect:			
Yes	0.041	0.332*	−0.238
	(0.143)	(0.176)	(0.208)
Observations	614	302	312
*R*2	0.69	0.76	0.65
B: Childcare			
Baseline:			
No children or children, with care	0.298***	0.442***	0.161
	(0.074)	(0.102)	(0.106)
Differential effect:			
Children, no care	0.051	0.388**	−0.305
	(0.154)	(0.182)	(0.228)
Observations	614	302	312
*R2*	0.69	0.76	0.66

*Note:* Panel A of Table A8 displays the effect of having children on mental health during the COVID-19 pandemic. Panel B displays the effect of having no help with childcare on mental health during the COVID-19 pandemic. Column (1) to (3) each correspond to a regression of the PHQ-4 score on year indicators, individual fixed effects and interactions with the group indicator and the indicator for the survey year 2020. The PHQ-4 score has been standardized to have mean zero and standard deviation one. Standard errors are clustered on the individual level and in parentheses. * *p<*0.10, ** *p<*0.05, *** *p<*0.01

**Table A9.** Long-Term Differences in Mental Health Between Self-Employed Individuals and Employees

	(1)	(2)	(3)
	All	Female	Male
Baseline:			
Employees	0.272***	0.316***	0.203***
	(0.021)	(0.029)	(0.029)
Differential effect:			
Self-employed	−0.056	0.097	−0.175
	(0.076)	(0.104)	(0.107)
Observations	6420	3834	2586
*R*2	0.68	0.67	0.70

*Note:* Table A.9 displays the changes in mental health for different employment states between 2019 and 2021, excluding the year 2020. Since not all observations from the first wave in 2020 are observed in the second wave 2021, the number of observations somewhat decreases. Column (1) to (3) each correspond to a regression of the PHQ-4 score on year indicators, individual fixed effects and interactions with the group indicator and the indicator for the survey year 2021. The PHQ-4 score has been standardized to have mean zero and standard deviation one. Standard errors are clustered on the individual level and in parentheses. * *p<*0.10, ** *p<*0.05, *** *p<*0.01.

## Appendix B The Self-Employed Versus Managers

In the *Main Results* section, we use employees as a control group to isolate the effect of the pandemic on the mental health of the self-employed. However, comparing the self-employed to the entire population of employees may raise the question to what extent the two groups are indeed comparable. As explained in the *Empirical Strategy* section, we address this issue by partialing out individual fixed effects, thus accounting for permanent differences between self-employed and organizationally employed individuals. As described in this section, we refine this approach by limiting the control groups to employees who likely have a job profile that is more like that of the self-employed: managers. Specifically, we include only those employees who were “engaged in highly skilled activities,” held a “managerial function,” or had “extensive managerial duties.” Using this narrower definition for our control group, we replicate the analysis from the *Main Results* section. Considering our empirical strategy, we would expect results that are different from those in our main analysis only insofar as the latter were driven by heterogeneity in mental health changes within the larger population of employees.

Inspecting Figure B4 and the corresponding estimates in Table B10 , we observe that the differences in mental health changes among women decrease in magnitude and significance, yet remain large.^20^ Similarly, the results in Figure B5 and Table B11 confirm our pattern of results from the *Main Results* section.

**Table B10.** Differences in Mental Health Between Self-Employed Individuals and Employees in Managerial Positions

	(1)	(2)	(3)
	All	Female	Male
Baseline: Managers	0.289***	0.353***	0.234***
	(0.038)	(0.060)	(0.048)
Differential effect: Self-employed	0.010	0.177	−0.145
	(0.082)	(0.113)	(0.115)
Observations	2154	1006	1148
*R*2	0.65	0.66	0.64

*Note:* Table B.10 displays the changes in mental health for different employment states between 2019 and 2020. Column (1) to (3) each correspond to a regression of the PHQ-4 score on year indicators, individual fixed effects and interactions with the group indicator and the indicator for the survey year 2020. The PHQ-4 score has been standardized to have mean zero and standard deviation one. Standard errors are clustered on the individual level and in parentheses. * *p<*0.10, ** *p<*0.05, *** *p <* 0.01.

**Table B11.** Differences in mental health between different groups

	(1)	(2)	(3)
	All	Female	Male
Baseline: (1) Managers with income loss	0.513***	0.482***	0.534***
	(0.118)	(0.180)	(0.156)
Differential effect: (2) Managers without income loss	−0.257**	−0.146	−0.350**
	(0.124)	(0.191)	(0.163)
(3) Self-employed with income loss	−0.195	0.117	−0.550**
	(0.163)	(0.222)	(0.240)
(4) Self-employed without income loss	−0.236	−0.066	−0.349*
	(0.146)	(0.226)	(0.191)
*p*-value (3)–(4)	0.771	0.332	0.346
Observations	2154	1006	1148
*R*2	0.65	0.66	0.64

*Note:* Table B.11 displays the effect of being in different employment states on mental health during the COVID-19 pandemic. Column (1) to (3) each correspond to a regression of the PHQ-4 score on year indicators, individual fixed effects and interactions with the group indicator and the indicator for the survey year 2020, respectively. The PHQ-4 score has been standardized to have mean zero and standard deviation one. Standard errors are clustered on the individual level and in parentheses. * *p <* 0.10, ** *p <* 0.05, *** *p <* 0.01.

==========
